# The 24-hour movement behaviour compositions of children with and without impaired motor coordination: The Moves-UP project

**DOI:** 10.1371/journal.pone.0319094

**Published:** 2025-02-25

**Authors:** Nils Swindell, Chelsea Starbuck, Siqi Jin, Harriet Barker, Gemma Thomas, Jimena Rueda-Hernandez, Catherine Crosby, Claire Barnes, Huw Summers, Gareth Stratton

**Affiliations:** 1 Applied Sports Technology Exercise and Medicine Research Centre, Swansea University, Swansea, Wales, United Kingdom; 2 Community Paediatric Physiotherapy and Occupational Therapy Department, Hafan Y Mor Children’s Development Centre, Singleton Hospital, Swansea Bay University Health Board, Wales, United Kingdom; 3 Department of Biomedical Engineering, Swansea University, Swansea, Wales, United Kingdom; Hong Kong Metropolitan University School of Nursing and Health Studies, HONG KONG

## Abstract

The 24-hour movement behaviours, including sleep, sedentary behaviour (SB), and moderate-to-vigorous physical activity (MVPA) are crucial for a child’s healthy growth and development. Yet, the full 24-hour movement behaviour composition has not been thoroughly explored in children with suspected Developmental Coordination Disorder (sDCD). The aim of this study was to compare the 24-hour movement behaviour compositions of children with sDCD to their typically developing (TD) peers and to assess the associations between movement behaviours and motor competence. Sixty-nine children (mean age 8.6 ±  1.6 years, 55% boys) wore a wrist-mounted accelerometers for seven consecutive days, completed a dynamic motor competence assessment and were screened for sDCD using the Developmental Coordination Disorder Questionnaire. Results of the compositional Isotemporal-substitution analysis indicated that children with sDCD spent less time in light physical activity (LPA) and MVPA and more time in SB compared to the TD group. No significant differences were observed during school or weekend periods. However, increasing MVPA in place of lower-intensity activities was associated with theoretical improvements in motor competence. The 24-hour movement behaviour profiles of children with sDCD identified in this study may have adverse implications for their future health and wellbeing, emphasising the need for targeted interventions, particularly during out-of-school hours.

## Introduction

Sufficient physical activity (PA) and sleep, combined with limited sedentary behaviour (SB) is essential for children’s physical, cognitive and social-emotional development [1]. In school-age children, motor competence has been recognised as an important enabler of PA and predictor of consequent health outcomes such as cardiorespiratory fitness, and adiposity [2]. Indeed, several systematic reviews have demonstrated a positive association between motor competence and PA [3,4]. However, most studies have examined the effects of motor competence on PA, even though the relationship is considered reciprocal [3]. According to the conceptual development model of Stodden et al [3], motor competence plays a dynamic and synergistic role in the uptake, maintenance, or decline of PA.

During early childhood (2–5 years) engagement in PA facilitates the development of motor skills. At this developmental stage, children’s perceived and actual motor competence are not well aligned and are only weakly associated with PA [4,5]. As self-awareness increases with age, perceived and actual motor competence become more aligned during middle childhood (6–9 years)[4] and become predictive of PA [5,6]. Therefore, actual and perceived motor competence can lead to a positive or negative trajectory of PA resulting in either continued PA engagement and motor skill development or a spiral of disengagement. Although there is growing evidence to support the Stodden model [3], few studies have considered PA within the composition of movement behaviours sleep, SB, light intensity activity (LPA) and moderate-to-vigorous physical activity (MVPA) that make up a 24-hour day. Compositional data analysis has emerged as the preferred method to assess associations between PA and health outcomes driven by the premise that PA behaviours over 24-hours are time-use data and that considering such behaviours independently may yield spurious inferences [7,8]. Findings from the compositional data analysis and isotemporal substitution studies have demonstrated associations between the combined movement behaviour composition and a range of health outcomes [9–11]. However, such studies emphasise that the role of one part of the compositional is only relative to the remaining composites, as any theoretical increase or decrease of time spent in one behaviour must result in equal time allocation in the remaining behaviours.

Evidence for the role of the movement behaviour composition in the development of motor competence has been inconsistent [12–14]. In primary age children (age 8.4 ± 1.8 years) substituting time, during school hours, from SB or LPA to MVPA results in positive associations with fundamental movement skills (FMS) [13]. In contrast, studies in preschool children suggest that time spent in SB may not be deleterious. For example, Martins and colleagues [14] counterintuitively demonstrated that FMS, specifically manipulative skills, (e.g., striking, bouncing, kicking and throwing), were improved when time during school, was reallocated from LPA to SB. Similarly, in a composition of weekend movement behaviours, increasing SB at the expense of LPA was positively associated with motor competence, while during the week, adding SB at the expense of MVPA was associated with improvement in motor competence [12]. These discrepancies may reflect important activities performed at low intensities that contribute towards the development of motor competence during the preschool years or the absence of clear associations between PA and motor competence which emerge in later childhood [6]. More research is needed to understand the relationships between movement behaviours and the development of motor competence in school-aged children, to inform future public health interventions.

In recent years, research has documented high rates of poor motor competence (30–77%) in school-age children which has been described as an epidemic of poor motor skills [15]. Besides, 5–6% of school-age children have Developmental Coordination Disorder (DCD) a neurodevelopmental disorder characterised by a marked impairment of motor competence [15], while many children with neurodiverse conditions including autism, attention deficit hyperactivity disorder and dyslexia also have poorer motor skills than their peers [16].

In the absence of effective interventions, children with low motor competence are likely to follow a negative PA trajectory [3]. However, little is known about how a decline in PA impacts the remaining movement behaviours such as sleep and SB and the implications for their continued growth and development. To our knowledge, no studies have compared the 24-hour movement behaviour composition of school aged children who are typically developing (TD) with those who have delayed motor competence or suspected DCD (sDCD).

The aims of this study were to: (1) investigate differences in the 24-h movement behaviour compositions (sleep, SB, LPA and MVPA) between children with sDCD and their TD peers; and (2) examine the strength of associations between movement behaviour compositions and motor competence.

## Materials and methods

### Participants and settings

This study is based on baseline data from the Moves-UP project, a school-based intervention aimed at improving motor coordination in primary school children with sDCD. The protocols for the Moves-UP project were approved by the Swansea University ethics committee (approval no: 3 2023 6474 6381) and written informed consent (Parents and schools) and assent (children) were obtained from all participants prior to data collection. Children from four schools in Swansea, South Wales, participated in this study. Data was collected between the 1st of November 2023 and the 30^th^ of January 2024 from a convenience sample that included sixty-nine children (55% male, aged 8.6 ± 1.6 years).

### Measurements

#### Movement behaviours.

Participants were asked to wear an Axivity AX3 (Axivity Ltd, Newcastle, UK) accelerometer on the non-dominant wrist 24 hours per day for 7 days with accelerations recorded at 100 Hz and a dynamic range of ±  8 g [17]. Data were downloaded using OmGui open-source software (OmGui v 1.0.0.43, Open Movement, Newcastle University, UK). All data were processed in R (http://cran.r-project.org) using GGIR v3.0.2 [18]. Signal processing included auto-calibration using local gravity as a reference [19] and the detection of non-wear. To convert raw triaxial accelerations into one omnidirectional measure of acceleration the Euclidean Norm minus one g, (ENMO) was calculated from raw accelerations from the three axes minus 1 g which represents the value of gravity $\text{ENMO }=\surd \left(x^{2}+y^{2}+z^{2}\right)–1$. ENMO values were averaged over 5-second epochs for further analysis [20].

Accelerometer non wear time periods were determined based on the standard deviation (SD) and value range of the accelerations at each axis, calculated for 60-minute windows with a 15-minute sliding window [20]. If 2 out of 3 axes had a SD <  13 mg or the value range was less than 50 mg, the time window was classified as non-wear [21]. Non wear data was imputed using the average at similar time points on other days of the week. Participants were excluded if less than four valid days of wear (i.e., at least 16 hours per day ^− 1^) were recorded [21] and/or if accelerometer post-calibration error was >  10 mg [19]. Child specific cut-points were used to define SB, LPA, and MVPA over 5-s epochs of averaged ENMO values ≤  50 mg >  50 mg and <  200 mg and ≥  200 mg, respectively [22]. Sleep duration was estimated using a polysomnography-validated algorithm employing its default settings and the heuristic algorithm looking at distribution of change in z-angle [23]. Average daily time at each intensity was calculated across the recording period and separately for weekdays, weekends and during school time for each participant.

#### Motor coordination.

Participants’ parents/guardians completed the Developmental Coordination Disorder Questionnaire (DCDQ+) [24]. Children aged 5 to 7 years and 11 months who scored below 47 (out of 75) and children aged from 8 to 9 years and 11 months who scored below 56 (out of 75) classified as “suspected DCD” (sDCD) for subsequent analysis. The DCDQ has dementated high internal consistency (>0.87) and sensitivity (70%–92%) when validated against the Movement Assessment Battery for Children assessment in a clinically referred sample [24]. Further, a recent systematic review and meta-analysis on the predictive validity of the DCDQ reported a sensitivity of 0.87 and a specificity of 0.83 demonstrating acceptable diagnostic accuracy for identifying children with motor coordination difficulties [25]. To mitigate against potential response bias, respondents were not given information of survey threshold or outcomes. Furthermore, teachers provided support in completing the questionnaire to parents/guardians who needed it.

#### Motor competence.

All children, regardless of DCDQ + score, completed The Short Form Dragon Challenge (SFDC) [26], an abbreviated version of The Dragon Challenge a valid, reliable, and dynamic measure of motor competence in children [27]. The SFDC comprises six tasks to assess three FMS: *Stability* (standing stalk, wobble spot), *Locomotion* (standing board jump, speed bounce) *and Object Control* (underarm throw and catch, alternate hand wall test). Children watched a demonstration of the SFDC and practiced each task in isolation before having a single attempt at the full assessment. The Dragon Challenge is a hybrid-based assessment of motor competence that uses equally weighted processes (quality of technique) and products (successfully achieving task goal), to provide an overall score out of 24 where a high score is indicative of a high level of motor competence. All assessments were conducted in situ by assessors who received standardised training to implement the SFDC assessment. The assessment has been shown to have good intra, interrater and test re-test reliability (r = 0.8), in a sample of children aged 10 ± 0.8 years from the UK [26].

#### Lifestyle behaviours.

Children completed the Child Health and Activity Tool (CHAT) [28], a self-report online survey, while under supervision of their teachers and research staff. CHAT has been used extensively in this age group and has shown acceptable validity [29]. The survey captures behaviours chronologically over a 24-hour period including typical screen use, how children spend their lunch break, whether they used active transport to school and access to opportunities for PA. The Welsh Index of Multiple Deprivation (WIMD), calculated from postcodes, was used as an indicator of deprivation [30].

### Statistical analysis

Conventional descriptive statistics and compositional data analysis was used to describe the study sample and movement behaviour variables respectively.

Compositional means were calculated as component-wise geometric mean of the observed time use variables (Sleep, SB, LPA and MVPA) as the measure of central tendency of compositional data and rescaled to sum up to 1,440-minutes for interpretation in minutes per day. The co-dependency/dispersion between movement behaviours was assessed using pair-wise log ratio variances between all behaviours and represented in a variation matrix. Geometric means bar plots were used to illustrate proportions of time spent in each behaviour, stratified by TD/sDCD group [31].

To enable standard statistical methods to be applied, the compositional data was transformed to real space coordinates using isometric log-ratio (ilr) transformation. Specifically, sequential binary partitioning was used to configure ilr coordinates allowing each movement behaviour to be assessed in relation to the remaining movement behaviours [8]. The four-part movement behaviour composition (sleep, SB, LPA, MVPA) were expressed as three ilr coordinates that captured the combined distribution of all parts of the composition.

To test if the mean composition of movement behaviours differed significantly between the TD and sDCD groups (aim 1), after controlling for sex, age and deprivation, compositional multivariate analysis of covariance (MANCOVA) models were fitted with the ilr coordinates as the dependant variable. Separate models were fitted for weekend, weekday, total (combined week and weekday) and school time compositions (08:45 to 15:15) To identify the movement behaviours responsible for significant group differences, bootstrap percentile confidence intervals for log-ratio differences between groups were produced [31]. To aid interpretation, log-ratio differences were back-transformed into percentages using the following formula $\left(\exp\left(\log ratio-difference\right)-1\right)*100$.

Multiple linear regression was used to investigate the relationship between each movement behaviour composition expressed as ilrs (explanatory variable) and motor competence (dependent variable) (aim 2). Covariates (age, sex and deprivation) were additionally included as explanatory variables. The significance of movement behaviour composition in each model was examined with the ‘car::Anova()’ function, which uses Wald Chi squared to calculate Type II tests. The ilr multiple linear regression models were further checked for linearity, normality, homoscedasticity, and outlying observations to ensure assumptions were not violated. The multiple linear regression models were used to predict differences in motor competence associated with the reallocation of a fixed duration of time between two activity behaviours, whilst the remaining behaviours were held constant. Predictions were repeated with incremental 5-minute pairwise reallocations (further details in supplementary material, S3 File). Compositional data analyses were conducted in R (http://cran.r-project.org) using the compositions and robCompositions packages. Alpha level was 0.05 for all analysis.

## Results

Of the 100 participants in the Moves-UP project, 84 had valid accelerometer data for at least four days while 81 returned a completed DCDQ. A total of 69 participants (55% boys), aged from 6.9 to 11.4 years old (M = 8.6 SD = 1.6) had valid accelerometer and DCDQ + data making them eligible for this study. There was no statistically significant difference between the age, sex or ethnicity of the included and excluded sample (all p > 0.05; supplementary material S1 Table). However, there was a significant association between having complete data and deprivation (*X*^2^ (3,100) = 18.28, p < 0.001). Post hoc analysis revealed that participants with complete data were more likely to be from the 2^nd^ and less likely to be from the 3^rd^ WIMD quartile respectively. Table 1 shows the descriptive demographic data of TD and sDCD groups.

**Table 1:** Comparison of demographic characteristics between TD children and children with sDCD.

	Total sample (n = 69)		TD (n = 39)		sDCD (n = 30)	
	Mean	SD	Mean	SD	Mean	SD
**Age (y)**	8.62	(1.6)	8.61	(1.8)	8.63	(1.4)
	**n**	**%**	**n**	**%**	**n**	**%**
**Sex (boys)**	38	[55]	21	[54]	17	(57)
**Deprivation (WIMD)**						
*1st quartile*	14	20.5	8	21.1	6	20.0
*2nd quartile*	35	51.5	19	50.0	16	53.3
*3rd quartile*	11	16.2	6	15.8	5	16.7
*4th quartile*	8	11.7	5	13.2	3	10.0
**Race/ethnicity**						
Asian	4	5.9	2	5.3	2	6.7
Black	1	1.5	1	2.6	–	–
Mixed	7	10.3	4	10.5	3	10.0
White	35	51.5	20	52.6	15	50.0
Prefer not to say	21	30.9	11	28.9	10	33.3

*TD* typically developing, s*DCD* suspected Developmental Coordination Disorder, *WIMD* Welsh Index of Multiple Deprivation.

The compositional means and percent of time spent in each component of the movement behaviour compositions are displayed in Table 2. Typically developing children spent on average, 30.3%, 48.0%, 17.0%, and 4.7% of their time in sleep, SB, LPA, MVPA, respectively. Children with suspected sDCD spent a greater proportion of their time in SB (51.1%) and less of their time in LPA and MVPA (14.8% and 3.7% respectively). Sleep time was similar for both groups.

**Table 2:** Compositional mean and percent of time spent in movement behaviours.

	Composition	Sleep		SB		LPA		MVPA	
		*Minutes (%)*		*Minutes (%)*		*Minutes (%)*		*Minutes (%)*	
**Typical** **Developing**	*Total*	436.3	(30.3)	691.7	(48.0)	244.6	(17.0)	67.4	(4.7)
	*Weekday*	435.1	(30.2)	683.5	(47.5)	252.3	(17.5)	69.1	(4.8)
	*Weekend*	465.3	(32.3)	699.6	(48.6)	215.6	(15.0)	59.5	(4.1)
	*School timey*	–	–	246.5	(63.2)	134.1	(29.1)	30.4	(7.1)
**Suspected DCD**	*Total*	437.4	(30.4)	735.6	(51.1)	213.7	(14.8)	53.3	(3.7)
	*Weekday*	430.7	(29.9)	736.1	(51.1)	218.1	(15.1)	55.1	(3.8)
	*Weekend*	489.1	(34.0)	706.4	(49.1)	197.0	(13.7)	47.5	(3.3)
	*School time*	–	–	257.5	(66.0)	104.6	(26.0)	27.9	(7.1)

*SB* sedentary behaviour, *LPA* light intensity physical activity, MVPA moderate-to-vigorous physical activity.

The variation matrices (supplementary material, S2 Table) containing all pair-wise log-ratio variances illustrate the overall variation within the compositions for the whole sample. A value close to zero implies that the time spent in the two respective behaviours is highly proportional. High proportionality suggests that there is a strong relationship or co-dependency. In contrast, large coefficients in the variation matrix indicate low proportionality between compositional parts. The largest variability between behaviours was observed for ratios of SB to MVPA and sleep and MVPA suggesting that time spent in MVPA was the least co-dependent on these two behaviours. The coefficients were also notably larger for the weekend composition further suggesting that MVPA was more co-dependant on SB and sleep during the week.

The movement behaviour compositions varied according to sex, age, ethnicity, and deprivation score (Supplementary material, S4 File). After controlling for these covariates, the MANCOVA analyses revealed significant differences in the total and the weekday composition between the TD and sDCD groups the above covariates (*F* (3, 63) = 2.734, p = 0.05) and (*F* (3, 63) = 3.00, p = 0.04) respectively.

**Fig 1:**
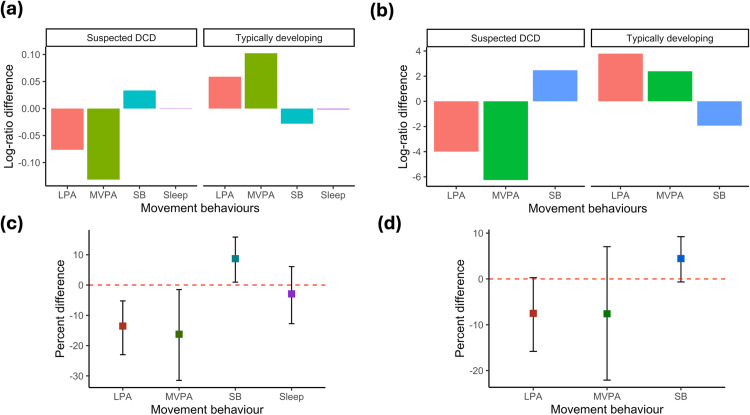
a and b compositional geometric mean bar plots comparing the compositional mean of the whole sample with the compositional mean of those with/without suspected DCD. c and d, the percentage differences, with bootstrap 95% percentile confidence intervals, in times spent in each movement behaviour between TD children and children with sDCD. a and c represent the combined weekday and weekend composition; b and d represent the school time composition. TD is the reference group meaning estimates represent differences for children with sDCD relative to the TD group.

Fig 1 illustrates that children with sDCD spent significantly less time in LPA and MVPA and more time in SB compared to the TD group. There was no statistically significant difference between the TD and sDCD group between the school time or the weekend composition (*F* (2, 52) = 1.60, p = 0.21) and (*F* (3, 63) = 0.34, p = 0.80) respectively.

The lifestyle behaviours for TD children and children with sDCD are presented in Table 3. Significantly more children with sDCD reported a preference to play alone at lunch time (23.1% vs 2.9%) and a greater proportion of children with sDCD were not part of a sports club outside of school (57.7% vs 20.6%).

**Table 3:** Comparison of lifestyle behaviours of TD children and children with sDCD.

	TD (n = 39)		sDCD (n = 30)		P (*X*^2^)
	n	%	n	%	
**Active transport to school**					
*Yes*	17	48.6	14	53.8	0.68
*No*	18	51.4	12	46.2	
**What did you do for your lunch break?**					
*Ran around*	21	60.0	16	61.5	0.09
*Sat around*	5	14.3	0	0	
*Stood around*	0	0	2	7.7	
*Walked around*	9	25.7	8	30.8	
**How many friends did you play with at lunch time?**					
*I like to play on my own*	1	2.9	6	23.1	**0.01**
*1–2*	8	22.8	9	34.6	
*3–4*	15	42.8	3	11.5	
*5 or more*	11	31.4	8	30.8	
**Can you walk to school from your home?**					
*Yes*	26	74.3	17	65.4	0.44
*No*	8	22.9	9	34.6	
**Can you walk to the park from your home?**					
*Yes*	31	88.6	20	76.9	0.22
*No*	4	11.4	6	23.1	
**Can you walk to a leisure centre?**					
*Yes*	12	34.3	6	23.1	0.22
*No*	23	65.7	20	76.9	
**Can you play in all the places you would like to?**					
*Hardly any of the places*	1	2.8	3	11.5	0.57
*A few of the places*	5	14.3	3	11.5	
*Some of the places*	19	54.3	12	46.2	
*All the places*	10	28.6	8	30.8	
**Do you have a garden?**					
*Yes*	31	88.5	25	96.2	0.28
*No*	4	11.4	1	3.8	
**How many times per week do you take part in a sports club outside of school?**					
*0*	7	20.6	15	57.7	**0.01**
*1–4*	21	61.8	9	34.6	
*5 or more*	6	17.6	2	7.7	
*TD* typically developing, sDCD suspected Developmental Coordination Disorder					

Linear regression models revealed that when expressed as ilr coordinates and adjusted for age, sex and deprivation score, the seven-day composition and weekday derived composition of movement behaviours significantly predicted motor competence (p = 0.003; r^2^ = 0.15) and (p = 0.006; r^2^ = 0.13) respectively. In contrast, the weekend derived composition and the school time derived composition were not associated with motor competence (p = 0.44; r^2^ = 0.01 and (p = 0.210, r^2^ = 0.016) respectively.

**Fig 2:**
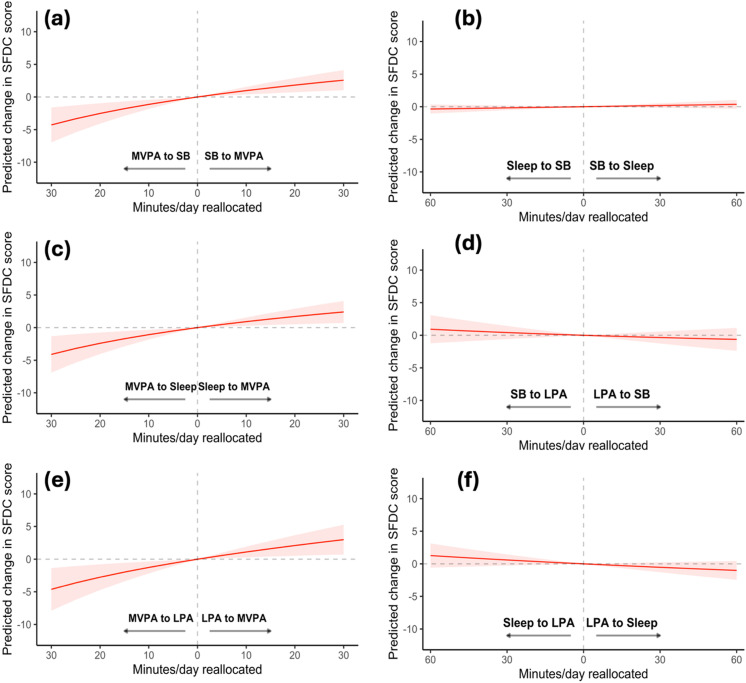
The predicted change in motor competence following the reallocation of time between specific movement behaviours, while holding the remaining behaviours constant. Reallocations between MVPA and SB a; sleep and SB b; MVPA and sleep c; SB and LPA d, MVPA and LPA e; sleep and LPA f.

Isotemporal substitution modelling results are presented graphically in Fig 2. Based on the 95% CIs, adding 5-minutes of MVPA at the expense of any behaviour predicted a significant improvement in motor competence (e.g., increasing MVPA at the expense of LPA predicted a 0.56 (95% CI 0.15 to 0.99) unit increase in motor competence). Reallocating time between the lower intensity movement behaviours (SB, LPA and sleep) did not predict a significant change in motor competence.

## Discussion

The aims of this investigation were to compare the 24-h movement behaviour compositions between children with sDCD and their TD peers and examine the strength of associations between movement behaviour compositions and motor competence. Our results indicate that when considering the 7-day or weekday compositions, children with sDCD spent significantly less time in MVPA and LPA and significantly more time in SB than their TD counterparts. In contrast to the present study, a previous study in early childhood (mean age 4.94 ± 0.59 years) found no difference in the wake-time movement behaviour compositions of children at risk of DCD relative to their TD peers [32]. These contrasting findings may reflect the age of participant in the respective studies. Indeed, the relationship between PA and motor competence has been shown to strengthen with age [2]. Further, the conceptual model of child development [3] suggests that among children with poor motor competence, PA declines with age as self-awareness increases and peer comparisons lead to lower perceived motor competence and reduced engagement in active pursuits [4,6]. Using a compositional approach the present study demonstrates that in children of primary school age those with sDCD exhibit less favourable profiles of MBs than their TD peers. Evidence from longitudinal studies suggest that without appropriate intervention, these differences (i.e., poor motor competence and low levels of PA) will persist throughout childhood and into adolescence [33,34] with adverse implications for physical, social and emotional health and particularly motor development through the life course.

According to the Diagnostic and Statistical Manual of Mental Disorders, a diagnosis of DCD requires children to demonstrate motor skills significantly below the expected level for their age, despite having adequate opportunities to learn and practice these skills [35]. The data presented indicates that primary-age children with sDCD engaged in less MVPA and were less likely to participate in active after-school clubs compared to their peers. As a result, they have fewer opportunities to develop fundamental motor skills. This trend likely reflects a tendency for children with motor difficulties to withdraw from or avoid movement-based activities, such as sports and group games, where peer comparisons are common [35]. Fortunately, physical inactivity and limited opportunities to build motor skills are modifiable factors. Targeted interventions that encourage active participation and support the development of fundamental motor skills are needed to help arrest negative trajectories in physical activity levels and motor competence. Such efforts can also help identify children who may require further assessment and specialised support.

In the present study, no differences were found in the weekend or school-time derived compositions, between the TD and sDCD groups, indicating that out-of-school time during weekdays is a key period of the day when behaviours differ between the two groups. In contrast to our findings, research in a pre-school setting [14] and from China [36] have shown that children with higher motor competence are more physically active during school time. The findings from a pre-school setting are likely explained by greater discretionary time available for PA relative to primary school. In Chinese schools, the emphasis on completing academic work outside of school makes school hours an important time for PA. Structured exercise opportunities are typically provided during lunchtime, appealing more to children with higher motor competence [36]. This dynamic may explain the observed association between motor skills and PA in Chinese educational settings.

None the less our findings concur with research from the UK which suggests that the greatest difference between the most and least active children occurs out of school, likely due to greater discretionary time available for PA [37]. In the UK, childhood obesity policy recommends that schools should deliver at least 30-minutes of the total recommended 60 daily minutes of PA for children [38]. Data presented here demonstrate that average time spent in MVPA at school was similar for both the TD and sDCD group at roughly 30-minutes. The similarity between the TD and sDCD groups at school may reflect a ceiling to the available time for PA during the school day and efforts by school staff to encourage children with sDCD to participate during physical education. However, it is important to note that although PA levels during school time were equivocal between the two groups, PA accumulated at school represented a greater proportion of total PA for those with sDCD. Thus, schools make an important contribution to children’s overall PA especially for those who are less active outside of school such as children with sDCD.

Middle childhood represents a time when many children begin taking part in active after school clubs, such as organised sport, that require increasingly complex motor skills. In the UK, ~ 80% of primary school aged children attend an after-school sports or exercise club on a weekly basis [39]. Children who attend after school clubs accumulated significantly more daily MVPA than children who do not [39]. Data presented here show that significantly fewer children with sDCD (42%) participate in after school clubs compared to TD children (79%). Research indicates that UK after-school programs for primary children predominantly feature team sports like football and rugby, hockey and netball, with insufficient emphasis on non-competitive physical activities [40]. Consequently, after school PA opportunities are more accessible to those with more advanced motor skills and confidence. Thus, increasing the number and variety of activities that children can attend may provide an effective means of increasing MVPA and subsequently motor competence in children sDCD [40,41].

When the seven-day and weekday MBs were treated as a composition, they collectively predicted motor competence after controlling for covariates. The isotemporal reallocation analysis further demonstrated that reallocating as little as 5-minutes to MVPA from any other movement behaviour while holding the remaining behaviours constant, predicted a significant increase in motor competence. These results corroborate findings from a recent longitudinal study by Estevan and colleagues [42] which found that among children aged 5 to 10 years achieving adequate levels of MVPA, at the expense of SB and LPA, was associated with increases in actual and perceived motor competence. Collectively these findings align with global PA guidelines which encourage children to be less sedentary and participate in more MVPA for their physical and mental health [1]. It is recommended that teachers, parents and practitioners should focus on increasing MVPA to support a positive trajectory of motor development and subsequent PA engagement.

Although the school setting has been shown to have a positive effect on children’s motor skills [43], our results found no association between the school time derived composition and motor competence. In a similar study with children of primary school age, Burns et al [13] found that for school time MBs, replacing SB and LPA with MVPA significantly predicted higher motor competence scores. Conversely, in a pre-school setting, only the reallocation of time from LPA to SB predicted enhancements in total motor competence and manipulative skills [14]. These disparities might be due to a more discretionary time in preschools compared to primary schools, the carryover of fine motor skills which occur at low movement intensities like drawing, writing, and cutting to object control skills like throwing and striking or age-related developmental changes. Indeed, characterising PA solely by movement intensity may be insufficient to discern its effects on motor competence, especially at lower movement intensities and activities requiring stability, balance and stillness. While increasing MVPA at the expense of lower intensity activities has been linked to better motor competence across various populations, ages, and settings [12,13,42,44], the impact of reallocating lower intensity movements has shown conflicting results [12,14,42,44]. Future studies should consider the types of activities children participate in, particularly during lower movement intensities, and their impact on motor skills.

This study has some limitations that should be acknowledged. First, the study was cross-sectional, therefore, the direction of causation is unknown. However, longitudinal research suggests that associations between motor competence and PA are reciprocal across childhood and into adolescence [34]. Therefore, motor competence scores are likely a cause and consequence of time spent in different movement behaviours. It is also impotent to emphasize that predictions made from linear models represent a theoretical level of motor competence for a given composition of movement behaviours and not an observed change in behaviour or associated outcomes. Second, only 69% of participants returned accelerometers with sufficient wear-time and completed the DCDQ. Although accelerator wear-time compliance was comparable to similar studies [45], non-adherence was negatively associated with deprivation indicating some selection bias. The relatively small sample size in this study increases the potential for Type-II errors. Consequently, insufficient statistical power may have contributed to the absence of a significant difference in MBs during the weekend and school time. Finally, we were only able to report on the combined SFDC score to represent motor competence. This approach may have masked associations between movement behaviour and individual FMS which have previously been shown to differ between specific FMS components [14].

None the less, this study has several strengths. To our knowledge, it is the first study in children of primary school age and only the second overall, to compare the 24-hour movement behaviour composition between TD children and children with sDCD, a particularly important group who are at greater risk of being inactive. The use of device-based assessment of MBs and the considerations of the combined 24-hour movement behaviour composite further strengthen findings from this analysis. Using compositional data analysis and isotemporal substitution recognises the compositional properties of daily behaviour and allows for meaningful and accurate inferences to be drawn without violating co-linearity assumptions. Furthermore, the separate consideration of weekday, weekend and the school time derived composition allows for contrasting assertions with motor competence and highlight specific times where positive changes in movement behaviour can be made. Finally, using a validated process and product orientated measure of FMS (SFDC) provides an assessment of motor competence equally weighted to quality of technique and execution (process) and the successfully achieving task goals (products).

## Conclusions

This is the first reported study to use a compositional data analysis approach to compare the 24-hour MB compositions of primary school children with impaired motor competence or sDCD and their typically developing (TD) peers. Our novel findings demonstrate that primary school children with sDCD spend significantly less time in MVPA and LPA and more time in SB than their TD counterparts. Furthermore, it highlights that increasing MVPA at the expense of activities of lower intensity was associated with a positive theoretical change in motor competence. Initiatives are needed to provide continued opportunities for PA and motor development, particularly in those with sDCD. Weekdays out of school time may be an apt time to increase PA participation in this population.

## Supporting information

S1 Table Comparison of included and excluded participants. (DOCX)

S2 Table Variation matrices presenting the variability of movement behaviour data in pair-wise log-ratios. (DOCX)

S3 File Description of Isotemporal substitution analysis. (DOCX)

S4 File Table differences in movement behaviour compositions between motor coordination groups and levels of sociodemographic factors. (DOCX)
