# Supplementary material for: The 24-hour movement behaviour compositions of children with and without impaired motor coordination: The Moves-UP project
Source: PLoS One. 2025 Feb 25;20(2):e0319094. doi: 10.1371/journal.pone.0319094 (PMC11856484; doi:10.1371/journal.pone.0319094)
Supplement: S1 Table — (DOCX) [file pone.0319094.s001.docx]

S1

**Comparison of included and excluded participants**

| S1 | **Included**  **(Complete data)**  **68** | | **Excluded**  **(incomplete data)**  **32** | | **t-test/qui squared test** |
| --- | --- | --- | --- | --- | --- |
| **Age (years)** | 8.6 | 1.6 | 9.1 | 1.9 | P = 0.219 |
| **Sex (boys)** | 37 | 54.5% | 19 | 59.4% | P = 0.641 |
| **Deprivation (WIMD)** |  |  |  |  | P = 0.001 |
| *1^st^ quartile* | 13 | 19.1% | 5 | 15.6% |  |
| *^2nd^ quartile* | 35 | 51.5%* | 4 | 12.5%* |  |
| *3rd quartile* | 11 | 16.2%* | 15 | 46.9%* |  |
| *4th quartile* | 9 | 13.2% | 8 | 25.0% |  |
| **Race/ethnicity** |  |  |  |  |  |
| Asian | 4 | 5.9 | 2 | 6.2 | P = 0.492 |
| Black | 1 | 1.5 | 2 | 6.2 |  |
| Mixed | 7 | 10.3 | 2 | 6.2 |  |
| White | 35 | 51.5 | 13 | 43.8 |  |
| Prefer not to say | 21 | 30.9 | 12 | 37.5 |  |

Table S1 presents the descriptives statistics for children with and without complete data.

participants with complete data were more likely to be from the 2^nd^ and less likely to be from the 3^rd^ WIMD quartile respectively.
