# Supplementary material for: The 24-hour movement behaviour compositions of children with and without impaired motor coordination: The Moves-UP project
Source: PLoS One. 2025 Feb 25;20(2):e0319094. doi: 10.1371/journal.pone.0319094 (PMC11856484; doi:10.1371/journal.pone.0319094)
Supplement: S2 Table — (DOCX) [file pone.0319094.s002.docx]

S2

Variation matrices ﻿presenting the variability of these data in pair-wise log-ratios to reflect the (proportional) relationship or co-dependence between two behaviours. A value close to zero suggest that the time spent in the two behaviours are highly proportional. In contrast, a high value lower co-dependency between two behaviours.

| **24-h movement behaviour composition** | | | | |
| --- | --- | --- | --- | --- |
| **Behaviour** | **Sleep** | **SB** | **LPA** | **MVPA** |
| Sleep | 0.00 | 0.25 | 0.27 | 0.29 |
| SB | 0.25 | 0.00 | 0.10 | 0.27 |
| LPA | 0.27 | 0.10 | 0.00 | 0.14 |
| MVPA | 0.29 | 0.27 | 0.14 | 0.00 |

| **Weekday composition** | | | | |
| --- | --- | --- | --- | --- |
| **Behaviour** | **Sleep** | **SB** | **LPA** | **MVPA** |
| Sleep | 0.00 | 0.25 | 0.26 | 0.28 |
| SB | 0.25 | 0.00 | 0.11 | 0.29 |
| LPA | 0.26 | 0.11 | 0.00 | 0.14 |
| MVPA | 0.28 | 0.29 | 0.14 | 0.00 |

| **Weekend composition** | | | | |
| --- | --- | --- | --- | --- |
| **Behaviour** | **Sleep** | **SB** | **LPA** | **MVPA** |
| Sleep | 0.00 | 0.16 | 0.15 | 0.36 |
| SB | 0.16 | 0.00 | 0.18 | 0.41 |
| LPA | 0.15 | 0.18 | 0.00 | 0.19 |
| MVPA | 0.36 | 0.41 | 0.19 | 0.00 |

| **School day composition** | | | |
| --- | --- | --- | --- |
| **Behaviour** | **SB** | **LPA** | **MVPA** |
| SB | 0.00 | 0.10 | 0.26 |
| LPA | 0.10 | 0.00 | 0.14 |
| MVPA | 0.26 | 0.14 | 0.00 |
