# Supplementary material for: The 24-hour movement behaviour compositions of children with and without impaired motor coordination: The Moves-UP project
Source: PLoS One. 2025 Feb 25;20(2):e0319094. doi: 10.1371/journal.pone.0319094 (PMC11856484; doi:10.1371/journal.pone.0319094)
Supplement: S3 File — (DOCX) [file pone.0319094.s003.docx]

S3. Isotemporal substitution

Multiple linear regression models were used to predict differences in motor competence associated with the reallocation of a fixed duration of time (5 minutes) between two activity behaviours, whilst the remaining behaviours were held constant. This was achieved by systematically creating a range of new activity compositions to mimic the reallocation of five minutes between all activity behaviour pairs, relative to the mean composition. The new compositions were expressed as ilr coordinate sets, and each subtracted from the mean composition ilr coordinates, to generate ilr differences. These ilr differences (each representing a 5-minute reallocation between two behaviours) were used in the linear models to determine estimated differences (95% CI) in motor competence.

Predictions were repeated with incremental 5-minute pairwise reallocations to a maximum of 60-minutes. This range was chosen to reflect what would be a viable actual change in 24-hour movement behaviours. Reallocations that included MVPA were limited to 30-minutes to avoid potentially distorted predictions from relocating a too great a proportion of the behaviour.
