# Supplementary material for: The 24-hour movement behaviour compositions of children with and without impaired motor coordination: The Moves-UP project
Source: PLoS One. 2025 Feb 25;20(2):e0319094. doi: 10.1371/journal.pone.0319094 (PMC11856484; doi:10.1371/journal.pone.0319094)
Supplement: S4 File — (DOCX) [file pone.0319094.s004.docx]

S4

**Compositional MANOVA of differences in movement behaviour compositions between motor coordination groups and levels of ﻿sociodemographic factors.**

| Table S4 |  |  |  |  |  |  |  |  |  |  |  |  |  | |  |  |  |
| --- | --- | --- | --- | --- | --- | --- | --- | --- | --- | --- | --- | --- | --- | --- | --- | --- | --- |
|  | **24-h MB composition** | | | | **Weekend MB composition** | | | | **Weekday MB composition** | | | | | **School day MB composition** | | | |
|  | *﻿Pillai’s trace* | *F* | *df* | *p* | *﻿Pillai’s trace* | *F* | *df* | *p* | *﻿Pillai’s trace* | *F* | *df* | *p* | *﻿Pillai’s trace* | | *F* | *df* | *p* |
| **sDCD/TD** | 0.115 | 2.811 | 1 | **0.046** | 0.016 | 0.343 | 3 | 0.7941 | 0.125 | 3.083 | 1 | **0.033** | 0.039 | | 1.328 | 1 | 0.217 |
| **Sex** | 0.185 | 6.126 | 1 | **0.001** | 0.192 | 6.450 | 1 | **0.001** | 0.155 | 4.956 | 1 | **0.003** | 0.107 | | 4.867 | 1 | **0.010** |
| **Ethnicity** | 0.148 | 0.910 | 4 | 0.538 | 0.109 | 0.663 | 4 | 0.785 | 0.165 | 1.016 | 4 | 0.436 | 0.229 | | 2.272 | 4 | **0.026** |
| **Age** | 0.053 | 0.697 | 2 | 0.65 | 0.102 | 1.369 | 2 | 0.230 | 0.049 | 0.644 | 2 | 0.695 | 0.058 | | 1.145 | 2 | 0.338 |
| **WIMD** | 0.039 | 0.965 | 1 | 0.416 | 0.006 | 0.165 | 1 | 0.920 | 0.051 | 1.27 | 1 | 0.290 | 0.098 | | 3.932 | 1 | **0.024** |

Table S2 presents the results from the MANOVA tests which were used to compare movement behaviour compositions between the motor coordination and sociodemographic groups. There were significant overall differences in the combined total movement behaviour composition and the weekday composition between sDCD and TD children (p ≤0.05) but not the for the school day or the weekend compositions (p ≥0.05).
